# Angiotensin-converting enzyme inhibitor activity of peptides derived from *Kacang* goat skin collagen through thermolysin hydrolysis

**DOI:** 10.14202/vetworld.2021.161-167

**Published:** 2021-01-21

**Authors:** Arby’in Pratiwi, Thoyib R. Hakim, Mohammad Z. Abidin, Nanung A. Fitriyanto, Jamhari Jamhari, Rusman Rusman, Yuny Erwanto

**Affiliations:** 1 Department of Animal Products Technology, Faculty of Animal Sciences, Universitas Gadjah Mada, Jl. Fauna No. 3, Bulaksumur, Yogyakarta 55281, Indonesia; 2 Division of Halal Materials Development, Institute for Halal Industry and System, Universitas Gadjah Mada, Jl. Kaliurang km. 4.5, Bulaksumur, Yogyakarta 55281, Indonesia

**Keywords:** angiotensin-I-converting enzyme inhibitor, bioactive peptides, collagen, hydrolysis, thermolysin

## Abstract

**Background and Aim:**

Angiotensin-converting enzyme (ACE) is one of the inhibitory enzymes isolated from animals for the treatment of hypertension. ACE inhibitor (ACE-I) peptides can be obtained by hydrolyzing proteins from various animal tissues, including muscle and connective tissues. However, the study on ACE-I activity from collagen of *Kacang* goat skin has not been conducted. This study explores the potency of collagen from *Kacang* goat skin as a source of an antihypertensive agent through ACE inhibition. Thermolysin will hydrolyze collagen and produce the peptide classified antihypertensive bioactive peptides. This study aimed to determine the potential of thermolysin to hydrolyze collagen of *Kacang* goat skin for ACE-I peptide production and to identify the production of ACE-I peptides.

**Materials and Methods:**

Collagen from *Kacang* goat skin was hydrolyzed with thermolysin and incubated at 37°C for 1 h. Molecular weight (MW) evaluation was performed by SDS PAGE; fractionation peptides at <5 kDa, 3-5 kDa, and <3 kDa were performed by ultrafiltration and ACE-I activity determined by IC_50_ measurement.

**Results:**

Collagen was hydrolyzed by thermolysin, resulting in protein with MW of 117.50-14.60 kDa. The protein content of fractionation at >5 kDa was 3.93±0.72 mg/mL, content of 3-5 kDa was 3.81±0.68 mg/mL, and that of <3 kDa was 2.33±0.38 mg/mL. Fractionation was performed 3 times and one of the results was selected for the ACE-I test. The selected fraction was tested by IC_50_ measurement with three repetitions and it showed an average enzyme activity at 0.83 mg/mL or 82.94 mg/mL.

**Conclusion:**

Thermolysin hydrolysis of collagen from *Kacang* goat skin showed the potential to produce bioactive peptides, such as ACE-I.

## Introduction

*Kacang* goat is one of the Indonesian local goat breeds and has spread throughout the country. The breed has a small body size. Furthermore, *Kacang* goat’s skin is commonly used as material for jackets and gloves manufacturers. However, its other potentials, such as being a source for collagen, have not been fully studied, even though the compound is abundant (constituting one-third of the body’s protein). Collagen functions as a cushion between cells and layers of tendons (supporting the skin and organs). Furthermore, it plays the role of a guardian of the shape and structure of the human body. It maintains and connects the delicate tissues in the skeleton. Collagen is the main structural component of white connective tissue, which covers almost 30% of the total protein in vertebrate and invertebrate organs. Collagen is a large molecular protein and a major component of the skin [1]. The hydrolysis of collagen produces potential bioactive peptides, such as angiotensin-converting enzyme 1 (ACE-I) compounds. ACE-I could reduce the risk of hypertension or high blood pressure and heart failure. High blood pressure, also known as hypertension, is a condition where the systolic blood pressure reaches ≥130-140 mmHg and the diastolic blood pressure reaches ≥80 mmHg [2]. The normal blood pressure of a normal height, weight, activity, and general health is 120/80 mmHg. According to the World Health Organization, the normal blood pressure is <130/85 mmHg, while blood pressure >140/90 mmHg is categorized as hypertension.

According to the results of primary health research in 2018, hypertension cases in Indonesia reached 26.5%, which was based on direct blood pressure measurement and disease history of the patients [3]. High blood pressure can lead to complications of various deadly diseases [4]. Hypertension can occur due to unhealthy lifestyle and dietary habits, including the consumption of fast processed foods with high fat, protein, salt, and low-fiber content [5]. At this time, the increase in hypertension sufferers exceeded 1 billion throughout the world [6]. Hypertension is a disease that can attack people’s health and cause death [7]. Research has shown that hypertension sufferers use synthetic angiotensin-converting enzyme inhibitors to cause several allergic diseases [8], hence the researcher’s effort to produce natural antihypertensive peptides. The antihypertensive peptide, which is known as ACE inhibitory peptide, is a polypeptide that can reduce blood pressure by inhibiting ACE [8]. Previous research has developed a method to obtain ACE inhibitor (ACE-I) from animal and vegetable proteins. The research showed that some peptides with antihypertensive effects had been successfully eliminated from all sorts of protein sources, including meat, soy, and fish, among others. Other researches had been conducted to obtain the peptides from chicken skin and claw [9], goat meat [10], cow skin [11], and cuttlefish [12]. There are three ways to obtain natural antihypertensive peptides: (1) Direct purification of all sorts of natural antihypertensive peptides from the organisms without hydrolysis), (2) through chemical (heating or acid treatment) or enzymatic (both direct and indirect hydrolyses by microorganisms) protein hydrolysis, and (3) through recombinant DNA technology combined with the chemical synthesis of active peptide. The bioactive peptide activity emerges in the form of hydrolyzed proteins from its natural form. The hydrolysis of natural proteins can be done with a wide range of protease enzymes, such as pepsin and chymotrypsin. Thermolysin is an enzyme derived from the metalloproteinases family of enzymes produced by the Gram-positive bacteria *Geobacillus stearothermophilus*. This enzyme is called a neutral proteinase enzyme due to its specific hydrophobic environment and peptide (amino endopeptidase) that will cleave the N-terminal L-Aspartyl-L-phenylalanine methyl ester bond. The hydrolysis can also be done using a trypsin enzyme, a protease enzyme that breaks the peptides into simpler compounds during hydrolysis. Both of these enzymes catalyze the hydrolysis of peptide bonds into simpler compounds, such as protease, peptone, polypeptide, dipeptide, and some amino acids peptide compounds. These simple compounds then can be further purified to obtain a compound with the highest ACE-I activity and then sequenced for identification.

The collagen extracted from *Kacang* goat skin has a potential to be the source of antihypertensive agent, as it has not yet been explored. Therefore, this study aimed to acknowledge the potential of collagen for ACE-I production by exploring the ACE-I activity to evaluate the antihypertensive activity of hydrolyzed peptides and to provide a theoretical foundation for the utilization of the animal by-products as well as the enhancement of natural blood pressure-lowering peptides from animal-based sources.

## Materials and Methods

### Ethical approval

No animals experimented in the present study. Thus, there was no requirement for ethical approval.

### Study period and location

The experimental study was conducted from August 2019 to June 2020. The study was carried out in the Laboratory of Leather, Animal By-Products and Waste Technology, Department of Animal Products Technology, Faculty of Animal Science, Universitas Gadjah Mada, Yogyakarta, Indonesia. Collagen preparation was extracted and lyophilized in Integrated Laboratory for Research and Testing, Universitas Gadjah Mada, Yogyakarta, Indonesia.

### Kacang goat skin

*Kacang* goat skin was obtained from Wonogiri, Central Java, Indonesia. The skin was extracted at the Department of Animal Products Technology, Faculty of Animal Sciences, Universitas Gadjah Mada, Yogyakarta, Indonesia.

### Collagen extract

*Kacang* goat skin extract was filtered using Whatman paper, precipitated by adding NaCl until a concentration of 2.6 M is reached, and mixed for a day at 20-25°C. Precipitation was done at 4°C by centrifugation at 4500 rpm for 30 min. Pellets were liquefied in 0.05 M acetic acid (ratio 1:5) of pH 3. Dialysis was performed using 20 mM buffer phosphate at pH 7.8 for 24 h with solution replacement after 12 h. The solution was cleansed with the distilled water, dried, and stored in the freezer before freeze-dried into collagen.

### Collagen hydrolysis

The collagen was hydrolyzed with thermolysin. A hydrolytic enzyme concentration of 0.1 U/mL was put into the collagen and incubated for 60 min at 37°C. The hydrolysis was stopped by heating at 85°C for 10 min and cooled on ice. The results were confirmed by SDS-PAGE.

### SDS-PAGE analysis

According to a modified method, SDS-PAGE of collagen was conducted before and after hydrolysis [13]. The process of electrophoresis began by preparing running buffer, sample buffer, Coomassie Brilliant Blue gel, destaining solution, 15% separating gel with 6.0 mL of 30% acrylamide, 3 mL of 1.5 Tris-HCl at pH 8.8, 0.12 mL of 10 % SDS, 2.77 mL of DD H_2_O, 0.42 mL of 4% acrylamide stacking gel, 0.35 mL of 1.5 Tris-HCl at pH 6.8, 0.005 mL of 10% SDS, and 2.228 mL of DD H_2_O. The solution was placed in a series of SDS-PAGE tool. Afterward, it was added 12 mL of soleplate gel, 100 mL of APS, and 10 mL of TEMED. The solution was then stirred until homogenous mixed, filled on a plate and coated with 0.4 mL of butanol, and allowed to stand for 30 min to condense. The solidified butanol layer was discarded and added a stacking gel solution over the separating gel. 3.0 mL stacking gel was poured with 40 μL APS and 4.0 μL TEMED, mixed, and filled on the glass disk. Between the glass disks, a well-forming comb was inserted for 15 min until the solution solidified. The molecular weight (MW) was measured by comparing the distance of protein fraction electrophoresis with markers. MW is calculated by the retardation factor of each band as follows: Retardation factor = “Range of protein movement”: “Range of color movement.”

### Hydrolysate collagen fractionation

The collagen hydrolysate was fractionated based on its MW with ultrafiltration centrifuge tubes at cutoff of <5 kDa, 3-5 kDa, and <3 kDa (Merck, Amicon Ultra, Centrifugal Filters). Three fractions (MW >5 kDa, 3-5 kDa, and <3kDa) were collected and approximately 2 mL of the remaining hydrolysates were transferred into >5 kDa cutoff and then centrifuged at 4000 rpm for 20 min until 0.5 mL trace solution is obtained. The filtrate with 3-5 and <3 kDa was then measured for protein concentration and analyzed for ACE-I activity.

### Protein concentration

Protein content was detected by Waddell method [14]. In brief, 10 μL sample fractions were mixed with 1 mL buffer + 5 μL sample. Waddell reagent (phosphate, NaOH, and CaCl_2_) was added and then the mixture was stirred for 2 min. The sample’s absorbance was calculated at 215 and 225 nm. The protein concentration was calculated using the following formula: Absorbance 215-Absorbance 225 × 28.8 mg/ml.

### ACE-I enzyme activity

The ACE-I activity was analyzed and measured based on Chusman [15] and Cheung [16] method. The synthetic peptide substrate used was Hippuryl-L-Histidyl-L-Leucine (HHL) (Sigma-Aldrich). As much as, 25 μL of the substrate (50 mM HHL in 0.1 M sodium borate buffer containing 0.3 M NaCl at pH 8.3) was added into 50 μL sample and incubated at 37°C for 5 min. The reaction was initiated by adding 18 μL of 0.02 U/mL ACE (Sigma, USA) and the mixture was incubated at 37°C for 5 min. The reaction was stopped by adding 250 μL 1 M HCl 0.1 N. The reaction’s product of hippuric acid (HA) was mixed with 1.5 mL ethyl acetate and shaken for 3 min. The mixture was centrifuged at 3600 rpm for 15 min. The supernatant was taken (1 mL), placed on a new tube, and dried at 100°C for 60 min. HA liberated by ACE was calculated spectrophotometrically at 228 nm using Shimadzu UV 1601 PC. The calculation of ACE inhibitor activity was determined by the ACEi (%): ACE inhibitor (%) = (Ec-Es)/((Ec-Eb)) × 100%.

Where, Ec = control absorbance

Es = sample absorbance

Eb = blank absorbance

### Inhibitory activity (IC_50_)

ACE-I activity was measured at half the maximal inhibitory concentration, which is often referred to as IC_50_. The inhibitory value was determined by an ACE-I curve based on the functional assays as amount of peptide used to overcome 50% of ACE activity.

### Statistical analysis

Analysis of variance was done using IBM SPSS Statistics for Windows, Version 24.0 (IBM Corp., Armonk, N.Y., USA) to analyze the data. Significant differences were tested by Duncan’s new multiple range test at 5% of the probability.

## Results

### Collagen MW profile

**Figure-1:**
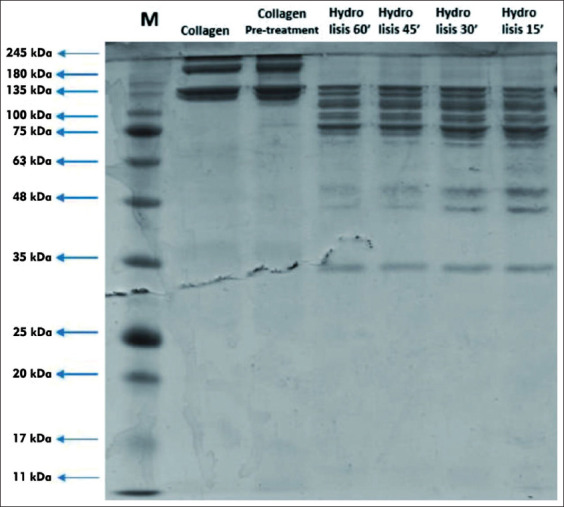
The protein molecular weight profile of hydrolysis of goat skin collagen by thermolysin for 60 min at 37°C.

The peptide molecular weight bands in collagen were affected by thermolysin hydrolysis until 1 h of incubation and the hydrolysis process was observed every 15 min, as shown in Figure-1. The detected peptide bands were at 227.99 kDa-17.76 kDa. Based on our preliminary experiment (data not shown), the optimum thermolysin activity on collagen occurred at around 37°C. According to SDS-PAGE analysis, 10 peptide bands after thermolysin hydrolysis at 130.81, 119.94, 109.96, 105.29, 84.75, 71.24, 38.80, 35.57, 32.61, and 17.76 kDa were detected after 1 h of incubation (Table-1).

**Table-1:** Data on MW calculation of collagen protein were hydrolyzed with the enzyme thermolysin (protein marker, band length, MW, log MW).

Protein marker (kDa)	Band length (cm)	Rf (x)	Log MW	MW (kDa)
245	0.01	0.02	2.35	227.99
180	0.25	0.04	2.26	185.11
140	0.65	0.12	2.11	130.81
	0.75	0.14	2.07	119.94
	0.85	0.15	2.04	109.96
100	0.90	0.16	2.02	105.29
	1.15	0.21	1.92	84.75
	1.35	0.25	1.85	71.24
35	2.05	0.37	1.58	38.80
	2.15	0.39	1.55	35.57
	2.25	0.42	1.51	32.61
10	2.95	0.54	1.24	17.76

MW=Molecular weight

### Log MW calculation of collagen

**Figure-2:**
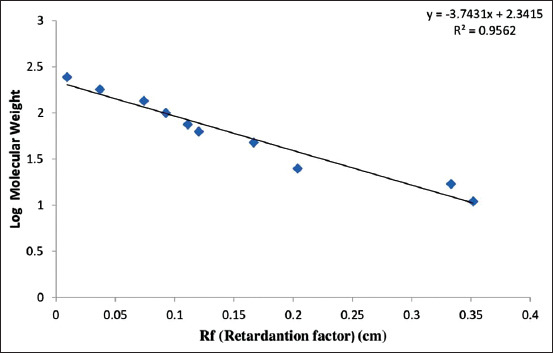
Equation curve between log value and Rf. symbols (+) represent Y. Linear regression equation was R^2^=0.96.

MW was measured using a standard curve, with y-axis indicating logarithm of MW and x-axis indicating division of the distance among the movement of the sample protein band, protein marker, or Rf (retardation factor). The results showed a linear equation: Y = −3.37x + 2.34, with R = 0.96, as shown in Figure-2.

### Fractionation of hydrolysate collagen and ACE-I activity

The result showed that, at fractionation of >5 kDa, ACE inhibition was around 72.72%. ACE-I activity in the fractionation of 3-5 kDa was 53.06% and ACE-I activity with fractionation of <3 kDa was 89.35% inhibition. The highest inhibitory activity was found at <3 kDa fractionation, while the lowest was found at 3-5 kDa fractionation, as shown in Table-2.

**Table-2:** ACE-inhibiting activity of hydrolysate from collagen after fractionation.

Fractionation types	Protein concentrations (mg/mL)	Protein in assay (µg/mL)	Inhibiting activity (%)
>5 kDa	1.20	8.13	72.72
3-5 kDa	1.00	8.47	53.06
<3 kDa	0.80	9.80	89.35

### Protein concentration

Protein concentrations of fractioned collagen (Table-3) were 2.40±0.05 mg/mL for 3 kDa ultrafiltration size. Furthermore, the fractionation of 3-5 kDa yielded a protein concentration of 2.95±0.65 mg/mL. For collagen fractionated by ultrafiltration with 5 kDa size, the protein concentration value was 3.69±1.12 mg/mL. The higher ultrafiltration size resulted in high protein content. Consequently, amount of the higher MW was found in the sample at 5 kDa ultrafiltration size. This phenomenon showed that the protein obtained by thermolysin enzymatic hydrolysis was more effective to produce proteins of >5 kDa MW. Protein measurements were conducted in three repetitions.

**Table-3:** The level of dissolved protein collagen hydrolyzed after fractionation (mg/mL).

Analysis of dissolved protein levels of collagen

MW fractionation	Protein concentrations (mg/mL)
<3 kDa	2.40±0.05^a^
3-5 kDa	2.95±0.65^ab^
>5 kDa	3.69±1.12^bc^

Different superscripts in the same column of protein content of MW fractionation by ultrafiltration indicate significant differences (p<0.05). MW=Molecular weight

### ACE-I activity

**Figure-3:**
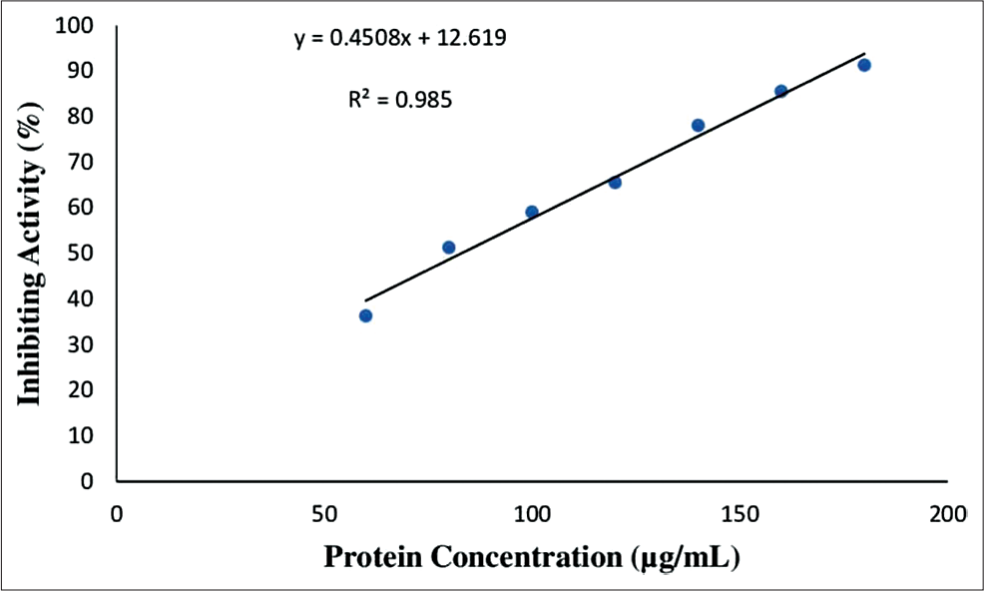
Linear regression curve of ACE hydrolyzed inhibition activity of collagen *Kacang* goat skin, symbols (•) represent point of ACE inhibition, while trendline (___) represents linear regression line from ACE inhibition. The results of linear regression equation: y = 0.45x + 12.61 and R^2^ = 0.98 for fractionation < 3 kDa.

The result shows the ACE inhibitory activity of *Kacang* goat’s skin collagen hydrolyzed with thermolysin and fractionated with <3 kDa membrane. The ACE inhibition activity ranged from 36.27% to 91.26% inhibition and the data are shown in Table-4. The IC_50_ value was recorded at 82.94 μg/mL protein concentration (Figure-3).

**Table-4:** ACE inhibitory activity of hydrolysis of *Kacang* goat’s skin collagen.

Volume inhibitor (µL)	Protein concentration (mg/mL)	Protein in assay (mg/mL) (x)	Inhibiting rating rate activity (%)
25	1.80	18.75	91.26±2.33^a^
25	1.60	16.66	85.53±5.23^a,b^
25	1.41	14.58	78.04±5.99^a,b^
25	1.20	12.50	65.53±9.41^b,c^
25	1.00	10.41	59.01±13.77^c^
25	0.80	8.33	51.32±17.17^a,c^
25	0.60	6.25	36.27±17.40^a,c^

Angiotensin-I-converting enzyme, different superscripts in the same column indicate significance (p<0.05) between samples

### Regression analysis for IC_50_ value

Linear regression was applied to calculate the IC_50_ of ACE inhibitory activity and the regression equation was y = 0.45x+12.61, with R^2^ = 0.98. The obtained inhibitory activity was used as a determinant for testing ACE inhibition and determining the IC_50_ value. The protein concentration corresponding to the IC_50_ value was 82.94 μg/mL. The IC_50_ value indicates the concentration of peptide required to inhibit 50% ACE, as shown in Figure-3.

## Discussion

### Collagen MW profile

Hydrolysis of the collagen produced several protein bands of various MW, as presented in Figure-1. Protein band after thermolysin hydrolysis in the collagen of *Kacang* goat’s skin had an MW value ranging from 130.81 to 17.76 kDa. Meanwhile, collagen (before hydrolysis) had MW of 227.99 kDa and MW of 185.11 kDa at pre-treatment. Thermolysin is a stable and specific enzyme that hydrolyzes protein at optimum temperature and time, thus affecting the hydrolysates’ MW and impacting the isolated bioactive peptides. Protein band during hydrolysis by thermolysin showed that the molecule weight after hydrolysis was lower than the MW before hydrolysis. This result was due to the enzymatic breakdown of collagen into peptides, amino acids, and other compounds [17]. The MW measurement in this research showed that the lower MW of collagen hydrolysate would migrate faster and had lower bands position. In contrast, larger molecules will migrate more slowly [18].

### Fractionation of collagen hydrolysate

Fractionation with membrane filtration at >5 kDa, 3-5 kDa, and < 3 kDa resulted in different MW and the ACE-I activity among the fractions of the collagen hydrolysate was also different. The result showed that < 3 kDa fractionation had the significantly highest (p<0.05) ACE-I activity. Many previous research identified active peptides at <3kDa [8,10,19,20]. Therefore, low molecular fractionation at <3 kDa was effective in isolating ACE-I peptides with the highest ACE-I activity. A similar result can be seen in research by Rubak *et al*. [21], who showed that <3 kDa MW fractionation on fermented milk supernatant had a higher ACE-I activity.

### Protein concentration

Protein separation by ultrafiltration showed that the value of protein content was largest at MW ≥5 kDa and that the low MW fraction after ultrafiltration was used for determining the bioactive peptide potency for ACE-1 testing. The higher protein content determined by ultrafiltration has been found at higher ultrafiltration size (more than 5 kDa); however, the protein obtained by enzymatic hydrolysis will be more efficacious [22]. Protein levels obtained were conducted by three repetitions and had significant (p<0.05) differences in terms of the ultrafiltration size.

### ACE-I activity and IC_50_

The ACE inhibitory activity with IC_50_ measurement was done to determine the potential of the peptide as an ACE-I. The high IC_50_ value indicates that the collagen hydrolysate of *Kacang* goat skin has the potential as an ACE-I. The IC_50_ was calculated by regression equations from dissolved protein content and inhibition percentage, as presented in Figure-3. Based on a previous study [19], the determination of inhibitory activity could be done using the regression line equation, where the x-axis indicates protein content and the y-axis indicates inhibitory percentage. A method that can be used in determining the inhibitory activity has been reported previously [20] using HHL. ACE-I could be detected by HHL by measurement of the HA release from HHL. This study showed that the ACE-I activity of hydrolyzed collagens at <3 kDa fractionation was in the range of 36.27-91.26%, with IC_50_ at 82.94 mg/mL. Lee *et al*. [20] measured the ACE-I activities of peptide derived from the duck skin by-product through enzymatic hydrolysis and fractioned into low molecular weight, the result showed the amino acid sequence of the ACE inhibitory peptide was identified as a hexapeptide Trp-Tyr-Pro-Ala-Ala-Pro, with a molecular weight of 693.90 Da. . HHL has the potential to be used for ACE-I activity measurement by determining its release [20]. ACE activity that does not lose amino acids will be high. However, if there is a termination of two amino acids, ACE will have a low inhibition value. The collagen hydrolysate using thermolysin has produced Met = methionine, His = histidine, Tyr = tyrosine, Ala = alanine, Asn = asparagine, Ser = serine, Thr = threonine, Gly = glycine, Lys = lysine, Glu = glutamate, and Asp = aspartate [23]. Meanwhile, the potential peptides from protein hydrolysis for ACE-I activity can be seen on its IC_50_ value [24].

## Conclusion

The collagen hydrolysates from *Kacang* goat skin produced bioactive peptides at low MW, as evidenced by certain low MW analysis. Moreover, the thermolysin hydrolysis also produced peptides with ACE-I activity above 50%. The collagen hydrolysate from *Kacang* goat skin is a potential source of bioactive peptides and can be utilized to improve human health.

## Authors’ Contributions

YE designed the experiment, supervised, and participated in the preparation and execution of the experiment, and in writing of the manuscript. AP and R conducted collagen extraction, sample preparation and collagen hydrolysis, peptide ultrafiltration and participated in writing the manuscript. JJ and RR conducted SDS-PAGE and ACE examination and participated in writing of the manuscript. NAF and MZA conducted the data analysis, ACE calculation and manuscript structure reviewing and language editing. All authors read and approved the final manuscript.
